# Designing Serious Computer Games for People With Moderate and Advanced Dementia: Interdisciplinary Theory-Driven Pilot Study

**DOI:** 10.2196/games.6514

**Published:** 2017-07-31

**Authors:** Chariklia Tziraki, Rakel Berenbaum, Daniel Gross, Judith Abikhzer, Boaz M Ben-David

**Affiliations:** ^1^ Melabev - Community Clubs for Eldercare Research and Development Department Jerusalem Israel; ^2^ Communication, Aging and Neuropsychology Lab (CANlab) Baruch Ivcher School of Psychology Interdisciplinary Center (IDC), Herzliya Herzliya Israel; ^3^ Department of Speech-Language Pathology Faculty of Medicine University of Toronto Toronto, ON Canada; ^4^ Rehabilitation Sciences Institute (RSI) Faculty of Medicine University of Toronto Toronto, ON Canada; ^5^ Toronto Rehabilitation Institute (TRI) University of Toronto Toronto, ON Canada

**Keywords:** serious games, dementia, functionality, learning in context, speed of processing

## Abstract

**Background:**

The field of serious games for people with dementia (PwD) is mostly driven by game-design principals typically applied to games created by and for younger individuals. Little has been done developing serious games to help PwD maintain cognition and to support functionality.

**Objectives:**

We aimed to create a theory-based serious game for PwD, with input from a multi-disciplinary team familiar with aging, dementia, and gaming theory, as well as direct input from end users (the iterative process). Targeting enhanced self-efficacy in daily activities, the goal was to generate a game that is acceptable, accessible and engaging for PwD.

**Methods:**

The theory-driven game development was based on the following learning theories: learning in context, errorless learning, building on capacities, and acknowledging biological changes—all with the aim to boost self-efficacy. The iterative participatory process was used for game screen development with input of 34 PwD and 14 healthy community dwelling older adults, aged over 65 years. Development of game screens was informed by the bio-psychological aging related disabilities (ie, motor, visual, and perception) as well as remaining neuropsychological capacities (ie, implicit memory) of PwD. At the conclusion of the iterative development process, a prototype game with 39 screens was used for a pilot study with 24 PwD and 14 healthy community dwelling older adults. The game was played twice weekly for 10 weeks.

**Results:**

Quantitative analysis showed that the average speed of successful screen completion was significantly longer for PwD compared with healthy older adults. Both PwD and controls showed an equivalent linear increase in the speed for task completion with practice by the third session (*P*<.02). Most important, the rate of improved processing speed with practice was not statistically different between PwD and controls. This may imply that some form of learning occurred for PwD at a nonsignificantly different rate than for controls. Qualitative results indicate that PwD found the game engaging and fun. Healthy older adults found the game too easy. Increase in self-reported self-efficacy was documented with PwD only.

**Conclusions:**

Our study demonstrated that PwD’s speed improved with practice at the same rate as healthy older adults. This implies that when tasks are designed to match PwD’s abilities, learning ensues. In addition, this pilot study of a serious game, designed for PwD, was accessible, acceptable, and enjoyable for end users. Games designed based on learning theories and input of end users and a multi-disciplinary team familiar with dementia and aging may have the potential of maintaining capacity and improving functionality of PwD. A larger longer study is needed to confirm our findings and evaluate the use of these games in assessing cognitive status and functionality.

## Introduction

### Background

Aging in place is a desirable social and economic goal in our rapidly aging global society [1]. Maintaining cognitive functionality while aging is important to achieve this goal. Cognitive stimulation games have been used and studied as a method for maintaining healthy aging brains [2]. The use of computer games for cognitive stimulation and prevention of cognitive decline in healthy older adults is a fast growing area of research, sometimes referred to as “neuro-games” [3,4].

A budding field of research is the use of computer games for people with dementia [5-9]. With the global rise of people with dementia (PwD) [10] and the huge economic cost of their care, there is an increasing desire to maintain PwD at home and not institutions, for as long as possible [11]. One of the key factors in keeping PwD in their homes, as opposed to nursing homes, is related to their ability to maintain functionality of simple daily activities, despite their cognitive decline. Indeed, when families opt for institutionalization, it is usually on the basis of a loss of the PwD’s ability to eat independently, as well as perform activities related to personal hygiene, such as grooming and toileting [12]. The development of modalities to maintain aging in place for PwD could include computer-based games specifically designed to accommodate functional limitations and build on their remaining capacities [13-15].

Serious games offer the promise of low cost interventions in the care of PwD [16]. In addition, they require minimal professional supervision (ie, by an occupational therapist) and can be played with the assistance of formal or informal caregivers. The American Society of Occupational Therapy has developed computer applications for assisting individuals with autism and dementia [17]. However, very few of the efforts cited have used theory-driven learning theories in the game development or reported on the iterative human centered design process of game development with the end users involvement.

This paper aimed to contribute to methodology of game design for PwD. Our goal was to create a serious game that is acceptable, accessible, and engaging for people with moderate and advanced dementia based on DSM-5 criteria [18]. Our approach aims to bridge the transfer gap between “game designers” practice and knowledge, and neuro-psychosocial scientific knowledge of aging and dementia. In addition, our game design considers theories of learning and the impact of the “built environment” as compensatory constructs in learning. The overall aim of our gaming approach was to facilitate people with moderate and advanced dementia to arrive at an increased sense of self efficacy, which, according to recent research in neuropsychology, directly contributes to psychological, cognitive, and physical health, and thus serves as a key enabler in exercising and prolonging functionality [19].

### Theoretical Framework for Game Screen Development

The game was designed with input from a multi-disciplinary team familiar with aging and dementia and gaming theory as well as direct input from end users (the iterative process) [20]. Each game screen was developed with the input of 34 PwD, 14 community dwelling healthy older adults (ages 65-90), an occupational therapist, gerontologist, an MD PhD specialist in technology for health, a computer engineer, and a PhD cognitive psychologist specializing in cognitive and sensory aging. The complete game includes 39 screens.

**Figure 1:**
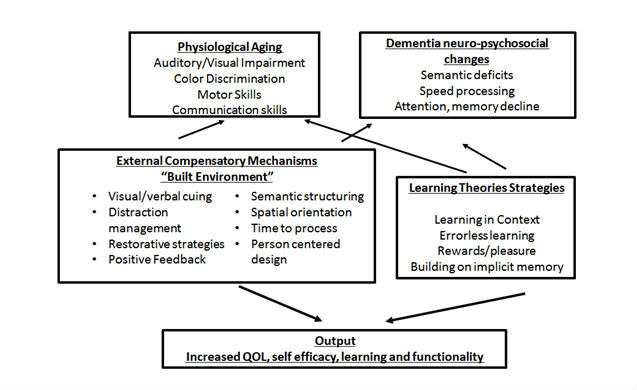
Multidisciplinary constructs and theories in designing serious games for people with dementia.

The theoretical models that form the underpinnings of our game are based on a multidisciplinary model outlined in Figure 1. The key frameworks involved (1) acknowledging the physiological changes associated with aging, (2) dementia’s neuropsychosocial induced changes, (3) applying learning theories that focus on “errorless learning,” learning in context, and building on remaining capacity (implicit memory), (4) external compensatory mechanisms, the “built environment” theoretical constructs including design, spatial orientation frames all brought to bear on, and (5) improving “self-perceived” self-efficacy. In later sections, each of these topics is briefly discussed first, and then the person-centered technological approach to the game development is presented, followed by the description of the iterative process of developing the game screens with direct input from the end users (people with moderate and advanced dementia).

### Enhancing Self Efficacy in PwD

The most important construct influencing our gaming strategy is aimed to enhance the self-efficacy of PwD, an important component of executive function [21]. A central problem that PwD experience is the gradual loss of cognitive and physiological capabilities in their daily lives. Indeed, not just intellectual tasks but simple activities of daily living (ADL) become more challenging. However, the literature shows (and experiential data in our daycare centers supports) there is a gap between a PwD capacity to learn and participate in daily tasks and their performance, as measured by cognitive instruments [22]. Physiological decline impacts on the PwD’s speed of completing tasks and movement. This is often exacerbated by family and caregivers who significantly and unknowingly contribute to PwD’s choice-limitations, as related to everyday living activities. Caregivers tend to do things for the PwD that the PwD could do on their own. This excessive involvement and over protection by caregivers tends to reduce the PwD’s confidence in their own abilities and competence, leading to premature disengagement by the PwD. On the other hand, adapted environments encourage independence in activity and help to maintain one's sense of perceived self-efficacy [23].

The concept of self-efficacy has grown out of a social psychology construct of human agency [24]. However, its bases are very old and embedded in such perennial philosophical underpinnings as theories of determinism, choice, intentionality, free will, and causality. There are 2 distinct, yet overlapping, theories that underlie the self-efficacy: (1) Motivational theories, which conceptualize self-efficacy in motivational terms and (2) Cognitive theories, which conceptualize self-efficacy in terms of expectancies and perceptions of control. Both theories, alongside empirical evidence, support the notion that self-efficacy plays a significant role in functionality (physical and cognitive) of PwD [25]. Therefore, our highest level objective in our design strategy was to utilize serious games to create the conditions and opportunities to rebuild and maintain a sense of self-efficacy, along with acknowledging the challenges on self-efficacy arising from normal and pathological physiological changes, as well as the PwD’s family and caregivers attitudes toward this slope of decline.

### Cognitive Changes Related to Aging and Dementia

One of the main characteristics of dementia relates to cognitive impairments, specifically, changes in memory encoding and memory retrieval. In addition, research supports that PwD also experience a reduction in executive functions—including planning, working memory, and selective attention [26]. Executive functions are central to most cognitive processes: the ability to focus on one aspect of the environment, to ignore other unrelated information, and to switch between them when prompted.

Selective attention has been marked as one of the major areas of cognitive impairments in dementia in general and Alzheimer dementia specifically [27], related to a reduction in the efficiency of inhibition [28], above and beyond age-related changes. This impairment may be linked with changes to frontal lobe regions [29]. These cognitive changes should be considered during game development. For example, reduced efficiency of inhibitory processes may translate to difficulties PwD will have in ignoring the irrelevant information presented on the screen during the game, or the information embedded in an irrelevant dimension of the stimuli presented (for a further discussion see Lustig et al [30]). Several aspects of our game were designed to tackle this change. For example, our design strategy was to avoid the clutter of the screen, thus reducing the amount of information PwD will need to inhibit. Additional factors related to the dementia process were taken into consideration, such as attention span, inhibition of initiation or perseveration, eye hand coordination, semantic sequencing, orientation to time and place, sustained attention, agnosia, and judgment.

### Sensory Motor Degradation Related to Aging and Dementia

Research shows that PwD do not face only cognitive deficiencies related to executive function, but also other deficiencies in auditory [31,32], visual [33] and other sensory systems [34] that contribute to cognitive deficits and difficulties in daily functionality [35]. For example, Ben-David and colleagues [36] have recently showed that reduced performance for PwD (as compared to healthy older adults) on a task that gauges executive functions (the Stroop color-word test) can be partially mediated by dementia-related changes in color-vision [37]. Auditory changes can also lead to reduced cognitive performance, especially in daily life activities such as communication [38]. This dual sensory loss (visual and auditory) also has direct implications on game administration. It reduces the comprehension of spoken instructions and increases the effort and the amount of cognitive resources invested in speech processing, thus tapping into the already reduced pool of resources [39-41]. Together, this cognitive and sensory interaction is expressed as a part of the information degradation hypothesis [42]. The theory postulates that as the perceptual system receives degraded information from the senses, it leads to reduced cognitive performance.

To address the above listed challenges, we considered multisensory approaches to enhance PwD’s daily functionality, such as using a variety of cues [43], both visual and auditory [44], as well as adjusting color and light setting. For example, an estimate of 88% of the aging population have very high failure rates of discrimination in the red-green and blue-yellow spectrum [45]. These age-related physiological changes were taken into consideration during the design relating to layout, color and instruction delivery methods and demonstration. Special attention in the design of the game was paid to the linguistics/semantic challenges of PwD [46-48].

Finally, sensory-motor degradation was considered in the design of the game environment. For example, during the iterative process, we learned from the comments of the end-users (34 PwD and 14 healthy community dwelling older adults) and the observations of the testers that the placement of the tablet has to be such as to allow visualization with natural light and no screen glare from artificial light or sun. The tablet should be placed in a comfortable position for the PwD, table height, and in a quiet environment with few distractions (again acknowledging cognitive changes).

### Making the Game Engaging

Serious games for older adults should be engaging and fun and further contribute to easing the personal burden of families and caregivers of PwD, as Robert and colleagues [49] among others, point out. The motivation to perform the task, an often-ignored factor, plays a large role in the performance of older adults. Specifically, framing tasks in an engaging, relevant context can improve performance [50]. For example, research by Zimerman et al [51] suggests that cognitive tasks, targeted originally with college students in mind, appear unsnagging for older adults, and may impact negatively on their ability to perform at their full capacity. This is of specific importance, as PwD are much more focused on emotional and social issues than on abstract problems [52-54]. While we aimed to design the serious game application in a simple “clean” fashion to facilitate sensory and cognitive processing, we were aware of the importance of designing the game screens in a visually engaging way. We postulate that when performing a task in an engaging context and by choosing stimuli that relate to PwD, the resulting increase in perceived self-efficacy would increase executive function and thus improve learning and performance. These relevant learning theories are discussed next.

### Learning Theories

The majority of serious games, or games for health, have utilized the important construct of entertainment as the major motivator for game construction. In our efforts to create a game for PwD based on information and communication technology (ICT), we put emphasis on age appropriate entertainment venues as defined by the end users themselves, and based on the concept that fun “learning in context” is a framework that induces capacity building for all persons and especially those people with disabilities, both physical and cognitive [55].

### Learning in Context

“Learning in context” has been defined in a variety of ways, however, the basic supposition is that adult learning does not take place in a vacuum, but within a sociocultural model, or as Hassin coined: learning “outside the mind” [56]. In the sociocultural models, learning is not something that happens, or is just inside the head, but instead, it is shaped by the context, culture, and tools in the learning situation. Russian psychologist LS Vygotsky was the pioneer of “learning in context”, a sociocultural theory of learning, in contrast to psychological and behavioral understandings of learning [57]. His work is based on the concept that all human activities take place in a cultural context with many levels of interactions, shared beliefs, values, knowledge, skills, structured relationships, and symbol systems [58]. These interactions and activities are mediated through the use of tools, either technical (machines, computers, calculators) or psychological (language, counting, writing, and strategies for learning), provided by the culture [59]. These tools ensure that linguistically created meanings have shared social meanings. His theories are relevant for our end-users, PwD, using technical and psychological tools to build upon the cultural learning of PwD and practice skills. Thus “learning in context” is a form of situated cognition [60]—that is, learning is inherently social in nature. Following this approach, learning takes place in 5 sequential phases that allow scaffolding of learning experiences (for a review, see [61]): (1) modeling, (2) approximating, (3) fading, (4) self-directed learning and, (5) generalizing.

Learning in context has been linked with basic cognitive constructs. Nisbett [62] postulated that implicit memory and learning is one of the products of context learning, based on the ontological assumption that interpretations of tasks are based on a background of past experience and intellectual resources. Nisbett suggested that cognitive structures are constructed and developed in particular social circumstances. The significance of cognitive structures resides in their deployment in cognitive activity, such as problem-solving, transfer, and learning.

Given the cognitive, physical, and sensory challenges of aging people with dementia, we focused on the above cited literature on learning theories to support our use of game screens, based on contextual learning. Specifically, our game screens utilized cultural memories and implicit memory, which are relatively more preserved for PwD. Implicit memory is one of the two main types of long-term memory which has recently been actively investigated as an important construct of cognitive function and overlooked to the usually measured explicit memory. Implicit memory includes procedural learning (eg, skills and habits), priming, and classical conditioning. These learning processes do not require conscious recollection of information, instead learning is expressed through performance or behavior [63]. Indeed, implicit memory or specifically non-declarative memory is acquired and used without the need (or ability) to verbally describe the process. For example, in procedural memory when tying one’s shoe or riding a bike, processes are learned and conducted without consciously thinking about the actions. It is a type of indirect, unintentional manifestation of prior experience [64].

Explicit memory, on the other hand, refers to the conscious, intentional recollection of factual information, previous experiences and concepts. While the literature documents well an age-related decline in explicit memory, numerous studies have shown that implicit memory is spared in older adults [65-67]. Even mild cognitively impaired older adults [68] and people with Alzheimer disease [69] showed some form of preserved implicit memory. This capacity can be utilized for reinforcing scaffolding learning theories. The aim of our game is to focus on practical activities in an entertaining, visually captivating and age appropriate presentation based on scaffolding learning theories [70].

### Errorless Learning

Within the framework of situated cognition learning in context, errorless learning methodology and cueing offers an important path to present the task so that a PwD overcomes inhibitions and limitations arising from low perceived self-efficacy. Errorless learning is “a teaching technique whereby people are prevented, as far as possible, from making mistakes while they are learning a new skill or acquiring new information” [71]. Major ways of achieving errorless learning are to use various cues, to complete the task collaboratively with the PwD, adjust the expectations of both client and designer, and make the task as doable as possible to the PwD. This approach assumes that new learning is stronger and more durable if mistakes are eliminated during training. Performance becomes automated through imitative learning and repetitive practice of perfect task execution. Errorless learning is not suited for all populations. With neurologically intact individuals, conscious or explicit memory of having made an error minimizes the impact of error learning. However, the deficit in explicit recall in PwD eliminates this counterweight to error learning and renders a PwD more vulnerable to its negative impact. In other words, PwD may remember the error, rather than learn the correct way to complete the task (ie, rather than learning that it was an error).

In the pertinent literature, there is an ongoing debate about the benefits of erroneous [72,73] versus errorless learning on memory creation. However, incorporating errorless learning scenarios within an active learning paradigm is a widely accepted practice in rehabilitation and dementia treatment, as it was found to maximize successful retrieval opportunities [74,75]. Indeed, errorless learning is taken as an encoding method that results in superior retrospective memory compared with erroneous learning. Neuropsychological studies indicate that people with compromised explicit memory are adversely affected by errors made during learning, and that implicit memory is sufficient to produce an errorless learning advantage for PwD [76]. This is perhaps due to the fact that erroneous learning demands greater frontal/executive contributions [77].

It is important to highlight the fact that there is something lost in an “errorless learning” approach. Psychological research in learning and memory identifies the opportunity to engage in difficult (hence error-prone) as very important in successful learning, most specifically for retrieval of learnt information (for a review, see [78]). However, working with PwD, we aim at compensatory learning approaches in an attempt to improve function by recruiting relatively intact neurocognitive processes to fill the role of impaired ones. Thus, it is assumed that new learning is stronger and more durable if mistakes are eliminated during training. Performance becomes automated through imitative learning and repetitive practice of perfect task execution [79].

In summary, all other factors being equal, it appears that there is ample evidence to suggest that errorless learning procedures are likely to improve retrieval in people with memory impairments relative to erroneous methods [80].

### Cueing, Priming, and Semantic Considerations

In addition to errorless learning in PwD, the procedure of cueing or priming and semantic structuring of instructions are important elements in cognitive functioning especially in semantic dementia. Priming is an implicit memory effect in which exposure to one stimulus (ie, perceptual pattern) influences the response to another stimulus [81]. The literature generally suggests that performance on implicit memory tasks, such as repetition priming, deteriorates in AD. However, these AD-related impairments were not found for all priming tasks. Indeed, in a longitudinal study using different priming tasks, only conceptual priming task (category- exemplar) was significantly impacted by AD neuropathology. Priming tasks that involves perceptual processing (word-identification, picture-naming, or word-stem completion tests) were not necessarily associated with a decline in AD [82,83]

Consequently, we chose in our game the use of visual-spatial cueing or priming [84]. Visual-spatial cueing represents a form of learning in context [85,86]. Using context to facilitate object recognition has gained importance in design, acknowledging both the role context plays in object recognition in human visual processing (Gestalt theory) and the striking algorithmic improvements that “visual context” has provided [87]. Based on the learning theories presented, we opted to use encouraging prompts when an error occurred. This method minimizes erroneous learning. Thus, it increases the impact of self-efficacy, building on the remaining capacities of a person to learn how to play the game successfully.

Special attention in the design of the game was given to the linguistics and semantic challenges of PwD, (for example, see [88,89]). These principles were incorporated in our game design by structuring the instructions in short simple sentences, for example, “Please drag the ball to the boy.” The modality of instructions delivery was also considered, in view of limitations in sustained attention, possible visual and auditory degradation, and cultural nuances of language. Therefore, in our game, instructions are provided in writing for each game screen, as well as vocal spoken instructions adapted to the culture of our target population. Every instruction for each game screen was tested with the end-users, (34 PwD and 14 healthy community dwelling older adults) various times during the iterative development process. Game screens were adapted and corrected for the final prototype game based on the verbal feedback of the end-users, as well as their ability to understand the instructions and succeed at the game as observed by the testers.

### Interaction of the Different Elements and Built Environment

We adopted modern viewpoints on cognitive performance in aging that consider the full context rather than focus on performance alone. In these views, all the elements of the model interact to shape performance. This complex interplay guides us in our design of the game and in our focus on human-centered technology, as discussed in the next section. For example, sensory changes were noted to affect performance on cognitive tasks in older age (sensory degradation hypothesis [90]), where reduced performance was linked with reduced acuity. Game engagement will clearly also be affected by sensory changes, as reduced sensory input leads to more effortful processing, potentially reducing engagement [91]. In other words, the game is less engaging if one cannot see it clearly. Learning in context is chosen to overcome cognitive changes in dementia, by using the most preserved intellectual abilities and knowledge [92]. Similarly, the choice of cueing and priming is designed considering visual sensory changes, and cognitive changes in dementia. Likewise, instructions and their presentation were designed considering learning in context, along with cognitive [93] and sensory changes.

This interplay can be exemplified in the variety of elements that are best classified as “built environment.” Built environment encompasses the design parameters related to the technological (machine) and screen design characteristics, as well as the physical environment within which the prototype game was pilot tested. In describing their CREATE model on designing technology for older adults, Rogers and Fink [94] explain that successful performance depends on demands imposed by the environment relative to capabilities of the individual (environmental press). This model illustrates the range and type of variables that must be considered when developing technology for older adults. As described in this introduction, our design methodology has taken many variables into consideration in order to develop a game best suited to PwD.

### Technology Considerations

In our overall strategy, we focused on person-centered technology, including the following 2 central guidelines: the Human Centered Design (HCD) and the Iterative Process [95,96].

The definition is outlined in the International Standardization Organization (ISO) standard Human Centered Design for Interactive Systems: ISO 9241-210 [97]. The HCD ISO guidelines are as follows: (1) Understand and specify the context of use, (2) Specify the user requirements, (3) Produce design solutions, and (4) Evaluate. We embedded this process within the iterative design process, where end-users (34 PwD and 14 healthy community dwelling elders) were involved directly in the creation and clarification of each game screen. The iterative, human centered approach [96] is the strategy we chose to follow for development of each game screen, as research shows that PwD, despite cognitive decline, can (and should) provide insight and user feedback that improves usability and human experience [98].

For example, at first we planned to use laptops, because we thought the portability would be convenient and the screen size would be appropriate for older adults. However, during the iterative development process, we learned from the end-users and observations of the testers that tablets were preferable, therefor the game development was switched from laptops to tablets. Tablets are easily mobile and can be easily disassociated from the keypad—a technology that often appears intimidating to PwD. Moreover, tablets use a touch screen and/or a stylus, an object resembling a pen, an element likely to be culturally more familiar to PwD then a keyboard. As we live in a society where technology is ubiquitous, our theoretical presupposition is that self-efficacy of PwD would be enhanced by their successful use of tablet technology [99,100].

### Game Framing Methodology

Broadly speaking, we developed a matrix based on the aforementioned theoretical frameworks that guided the creation of every game screen. A brief summary of these variables is depicted in Table 1. The aim was to create a fun and engaging game environment that is, on one hand challenging enough to provide an exercising and learning effect, while on the other hand, specifically adapted to assist in exercising key cognitive strengths a PwD has available (such as implicit memory), while providing assistive mechanisms to help overcome extraneous limitations (that would impede the accomplishing of tasks).

**Table 1:** Examples of variables taken into consideration for game screen frames.

Challenges	Variables	Solutions
Sensory degradation	Visual	Avoid blue or yellow combination, script choice
	Spatial placement of fields of action	Center of screen
Learning	“Learning in context” visual elements	Culturally relevant
	Cuing	Placing correct answer center screen, reminder by reading instructions over
	Feedback	Positively framed, immediate, errorless, and entertaining
Cognitive changes	Semantics	Simple action oriented instruction
	Uncluttered (inhibition)	No unnecessary information
	Technology complexity	Each time the game is played, it is preceded by practice exercises related to tablet use (ie, touch and drag functions). The practice exercises aren’t included in the analytics of the game session

We identified a set of functional simple daily tasks that are essential and culturally relevant to daily life. Each task was then divided into subtasks, utilizing an occupational therapy methodology, primarily adapted from neuro-rehabilitation [101]. Each subtask was further clarified in terms of the main key cognitive skills it reflects. While it is of course not possible to untangle different cognitive skills during task performance, it is possible to identify the main cognitive skills around which the game screen is designed, that is, executive function, eye hand coordination, working memory, and prolonged attention [102].

Each game screen was person-centered [103], and was designed in such a way that a measurement instrument collected game performance data (ie, speed of initial interaction with the game screen, speed of successful screen completion, and number of screens completed successfully).

**Figure 2:**
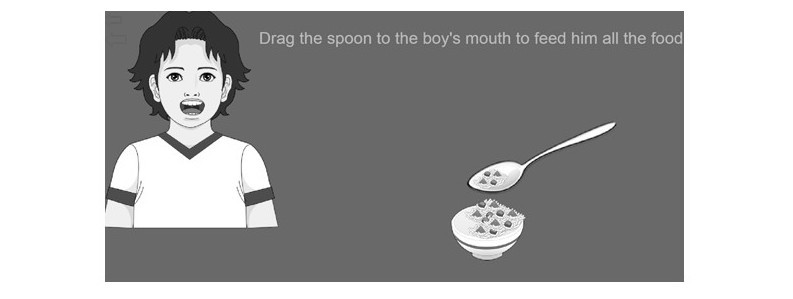
Sample game frame.

One sample game frame is presented in Figure 2. In this frame, the PwD was instructed to follow written and oral instructions to find, drag, and move items on the tablet touch screen. Table 2 describes the other various actions or tasks the PwD were asked to do in other game screens. It also lists the skills targeted by all of the game screens.

At the end of the iterative development stage, we had developed a prototype of a tablet-based game for PwD with 39 game screens. The prototype was used for the proof of concept pilot study that we report on next.

**Table 2:** Game screens: game types and skills involved. A list of the nine major game types used in the study, with all relevant physical and cognitive skills targeted.

Game types	Physical, cognitive skills targeted	Skills targeted on all games
1. Identify, find and touch	Gnosis	Eye hand coordination, language skills (reading, comprehension), understanding and following instructions, praxis, memory, sustained attention, and object recognition.
2. Identify, find and drag	Association, gnosis	
3. Identify, find, touch alternating correct answers	Mental rigidity	
4. Find, sort and drag	Gnosis	
5. Time orientation	Recognition, abstraction, association, match activity with time of day	
6. Space orientation	Recognition, gnosis	
7. Hold release action	Inhibition, basic math skills	
8. Drag things on screen into a sequence	Logic, executive functions	
9. Language exercises	Word finding, letter recognition, gnosis, semantic sequencing	

### Goals

The aim of our research was to answer the following questions: (1) Are serious computer games acceptable accessible and engaging for people with moderate and advanced dementia? (2) Are people with moderate and advanced dementia able to use a tablet? and (3) Can PwD improve the speed of performing a task with practice, indicating their ability to learn?

## Methods

### Procedure

A pilot study for proof of concept was conducted to answer the above questions. The game was played with the PwD and a tester present in a quiet room, located in the MELABEV dementia day center, Jerusalem, Israel. MELABEV has four day-care centers attended by approximately 500 PwDs, ranging from people with moderate cognitive impairment (MCI) to advanced dementia. MELABEV’s professional staff routinely uses computer games on a one-to-one basis for cognitive stimulation gaming [104], as well as reminiscence therapy at the computer [105]. Primary family caregivers who enroll the PwD in the day care program consent to the participation of their family member with these kinds of technology, as well as all other activities in the day care center.

Meaningful informed consent for people with dementia is challenging. Thus, for our pilot study, we utilized the participatory consent process [106]—each time a game was presented, the participant was asked by the tester if she agreed to participate in the gaming session. Upon agreement, the PwD voluntarily got up and was guided to the designated space – the computer room, to play the game. If the PwD did not agree to participate, he/she remained in the regular activity room, did not go to the computer room and did not use the game that time, with no consequences what so ever to the services they received in the center. If at any time during the game session, the PwD said or acted as if they didn't want to continue, the game session was terminated and they were taken back to the regular activity room.

During the 10 week pilot study, the PwD played the prototype game 1-2 times a week under supervision of testers. There were 6 different testers. All testers had past experience working with the PwD population: occupational therapist, gerontologist, social worker, pre-med student, occupational therapist student, and activity worker. Only 2 of the 6 were involved in the development of the game.

Testers’ main task was to observe the sessions and manually record their observations related to the PwD’s interaction with the game for each game frame. They also recorded unsolicited, unprompted spontaneous verbal comments made by the PwD while using the game. Also, testers assisted PwD to maintain their attention on the game throughout the session by prompting them to refocus, when this was called for. Finally, testers were instructed to assist with any technological issues that might arrive.

Each game session was between 20-30 minutes, a recommended time for therapy sessions with PwD. All sessions took place at approximately the same time of day in a quiet room. In every game session, each PwD had the opportunity to play the complete game of 39 game screens. Each game screen was played in the following way. If they were successful, they received a success message (audibly and visually) relevant to the activity performed. If the PwD did not succeed at first, they were cued (audibly and visually). The cueing procedure repeated 3 times, and then, even if the person didn't complete the screen successfully, the game advanced to the next screen. Success or failure, as well as other variables were recorded internally by the tablet.

### Participants

Out of about 200 PwD from two of MELABEV’s day care centers with moderate to advanced dementia, 24 persons were found to fit the inclusion criteria and participated in the pilot study (age range: 65 years – 90 years, 15 women, and 9 men). The PwD included had cognitive assessment scores (as tested by the Montreal Cognitive Assessment MoCA) as low as 6/30 [107] or a Mini-Mental State Examination (MMSE) as low as 10/30 [108]. We excluded patients with aggression, delusional behavior, a history of alcohol or substance abuse, depression, severe auditory, and visual or motor deficits, as assessed by the professional staff at MELABEV.

Fourteen healthy community dwelling older adults (age range: 65 years – 90 years; 11 women, 3 men) also volunteered to participate in this process. Game sessions took place in their homes at the time that was convenient for them. These older adults served as an age-matched control group and could verbalize their opinions relating to the games accessibility and acceptability better than PwD.

### Analysis

A mixed methods approach was utilized for evaluation [109]. Quantitative data for each participant was recorded automatically by the tablet platform, collecting game performance data on speed of successful screen completion and task completion rate. These data were analyzed using a mixed-model repeated-measure ANOVA (analysis of variance).

Qualitative data included the observations of the 6 testers from each game session they participated in, as well as the spontaneous comments from participants during the game session. The testers recorded their observations and the participant’s comments relating to each game screen in an Excel document immediately after each game session. The Excel (Microsoft) document was analyzed for themes using grounded theory by 2 researchers and a research assistant, each one separately. Analysis was then discussed as a group between the 3 researchers until consensus about common themes was reached. A list of 10 themes emerged. One of the major themes relates to self-efficacy of PwD and is discussed in this paper. Other themes will be discussed in a future paper.

## Results

### Participants

Of the 24 PwD who began the pilot study, 12 (50%) dropped out during the study. Reasons for dropping out included: rapid deterioration of physical and/or cognitive condition, vision deterioration, did not attend day care center due to illness, institutionalization, death, preference of other programs going on in the activity room, lack of interest in the game, and found the game to be too easy. Of those that dropped out 3 (12.5%) were game related (too easy, didn’t interest them) and 9 (37.5%) were aging or dementia related.

### Analysis

**Figure 3:**
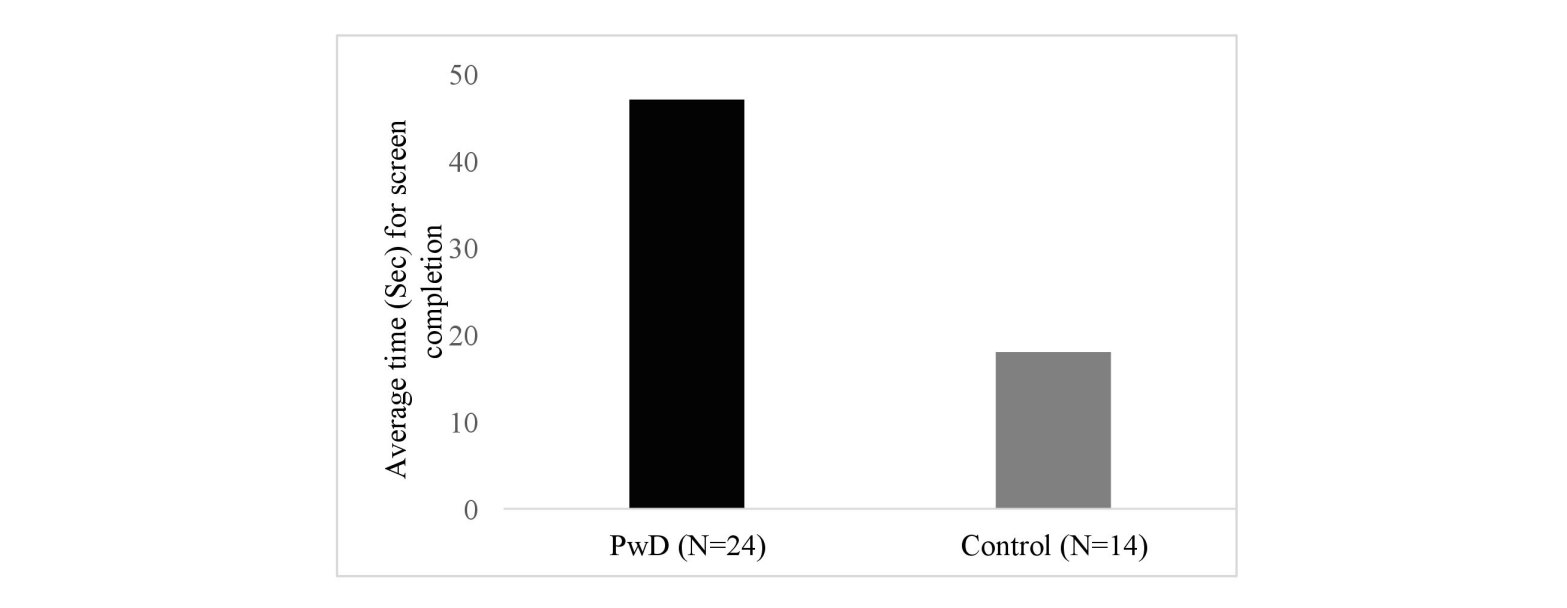
Average speed in seconds of successful screen completion for people with dementia and controls.

As expected, quantitative analysis showed that the average speed of successful screen completion was significantly longer for PwD compared with healthy older adults, *t*_34_=4.4, *P*<.001 (see Figure 3), with an average of 45.5 (SE 5.1) and 17.4 (SE 1.1) seconds/game frame for PwD and healthy controls, respectively. Note that, as expected, performance was much more varied across PwD than across controls.

**Figure 4:**
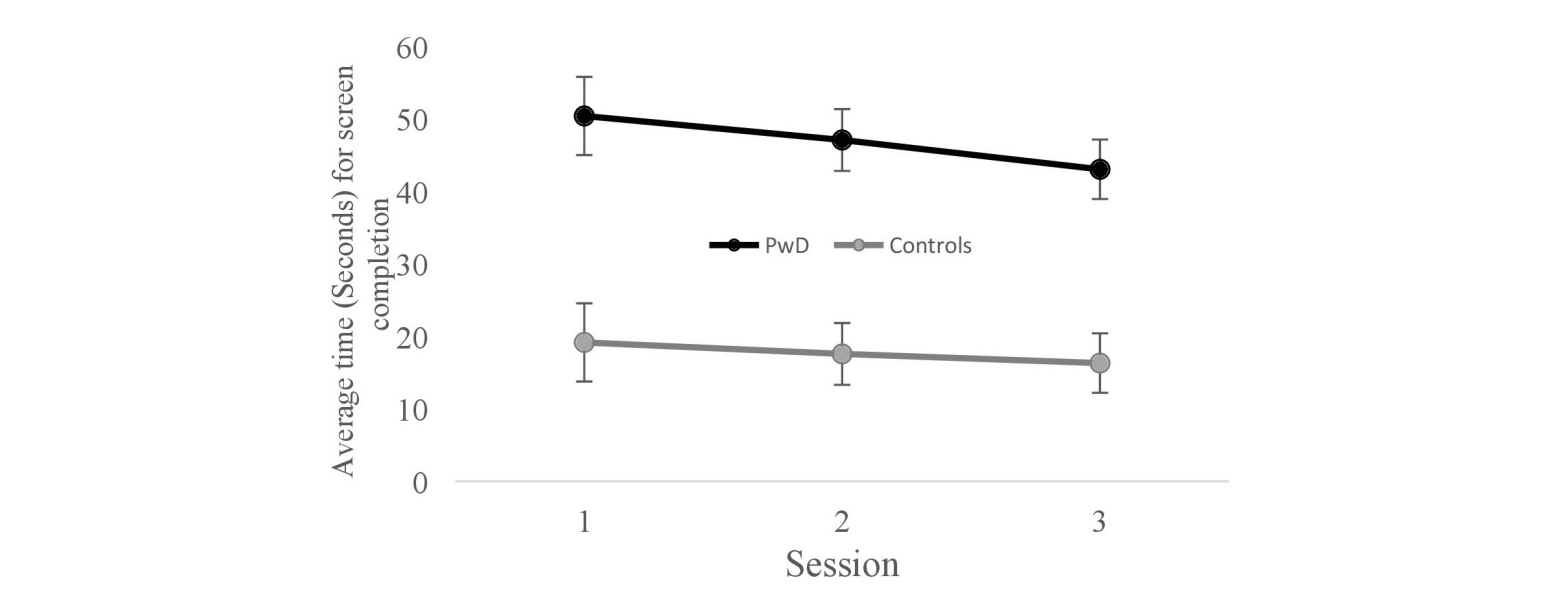
Average speed in seconds of successful screen completion for people with dementia and controls as a function of practice in the first three sessions.

Next, Figure 4 presents the average speed for successful screen completion for the first 3 sessions, separately for PwD and controls. To test whether performance improved with practice to the same extent for the two groups, a mixed-model repeated-measures ANOVA was conducted. Speed of screen completion was the dependent variable, session (1, 2, or 3) served as the within participants variable and group (PwD vs controls) as the between participant variable. A significant linear trend for session (ie, session 1 > 2 > 3) was found across both groups, *F*_1, 20_=6.1, *P*=.02, *η*_p_^2^=.23, denoting an increase in speed with practice. Clearly, a main effect for group membership was noted, with significantly slower performance for PwD than for controls, *F*_1, 20_=23.3, *P*<.001, *η*_p_^2^ = .54, but the linear trend did not interact significantly with group membership, *F*_1,20_=1.1, *P*>.3. In other words, the rate of improved speed with practice for PwD and healthy controls was not statistically different. Finally, the average number of game screens completed correctly by PwD per game session was 13.4 out of 22, representing 61% of the game frames.

In sum, these results may suggest that the tasks were well designed for the PwD group that is challenging enough to encourage improved performance, but not too challenging as to frustrate learning. For our control group, it appears that the tasks were easy and they quickly reached a ceiling of performance. Most importantly, it appears that when tasks are designed with PwD in mind, the rate of improvement in performance with practice (ie, learning) is not significantly different than the rate for healthy age-matched controls.

Qualitative analysis of the PwD spontaneous comments (eg, expressed while playing the game), as recorded manually by testers, reveal the following major themes in accessibility, acceptability, engagement, and self-efficacy.

First, it appears that the PwD were able to interact with the tablet and the game was acceptable to them and they even enjoyed playing it as indicated by the following:

“Thanks for choosing me to play the game.” C.

“I will recommend it to all my friends.” G.

“It was lovely.” C.

The enjoyment was not dependent on cognitive ability or on getting the correct answer. This was even the case with PwD who performed poorly on the game. For example, one woman would sing along with the game with a smile on her face even when she did not get the correct answer. Healthy older adults, on the other hand, found the game too easy, and on the most part not highly engaging.

In addition, we have some preliminary qualitative indicators that PwD’s self-efficacy was improved. Quotes from the PwD expressed a sense of self-worth and an increase in their self-esteem with the use of the game as the testers heard quotes such as

“I did it!” M.

“Now I know what utensil goes with what” M.

Increase in self-reported self-efficacy was found and seen with PwD only, and not reported by the healthy community dwelling older adults.

The PwD were able to remember certain game components, both those that were easy for them and those that were more difficult, as demonstrated from this spontaneous comment from a PwD to the tester accompanying him: *“I can play the game, except for one that is a bit harder.”* C.

We observed learning and special learning techniques used by the PwD in order to progress in the game. For example, one tester overheard the PwD speak to the tablet, which asked him for the answer for a second time saying, *“I know, I know, I am working on it.” C.* He expressed the fact that he was thinking and interacting with the tablet.

Testers observed that auditory cueing improved PwD’s performance and engagement with the game.

## Discussion

### Relevance of Our Findings

The field of serious games for PwD is in its infancy. Our paper reporting on a research and development project aims to add much needed initial knowledge in this area. In relation to our original research questions, we learned that: (1) serious computer games can be acceptable and accessible to PwD; (2) people with moderate and advanced dementia are able to use a tablet; and (3) PwD improved in their speed of successful screen completion with practice, at a non-significantly different rate than healthy older adults, implying some form of significant learning occurred (see Figure 4).

From qualitative analysis of PwD spontaneous comments, we learned that PwD enjoyed using the game. Our findings are consistent with previous research suggesting that technology can be empowering and satisfying to participants [110].

Although it is generally assumed that PwD cannot learn new information and skills, our exploratory data show that some of those who used the game learned how to do many of its activities. Future research will test exactly what is learned in the game, and more importantly, if there is a transfer of knowledge from the game to real life scenarios over time.

There are several additional key themes that emerged in this pilot study that may be useful for clinical intervention and future game design. First, from the observations of the occupational therapists it appears that PwD can use a tablet better than a laptop. It was found to be easier for them to manipulate [111], as they can adjust it and hold it with minimal difficulties. Indeed, the touch screen response mode is easier than a mouse or keyboard [112]. Second, the testers observed that auditory cueing improves PwD’s performance, supporting some of the findings in the literature [113-115].

Finally, it was encouraging to see that even people with dementia, who at the outset were hesitant to play the game, also had a positive interaction with the technology. Specifically, PwD who initially said that “this is not for me” because “I don’t know anything about tablets,” reported enjoying the game after their initial trial session and learning how to interact with it.

### Limitations

This initial exploration has several limitations. The sample size was small, the duration was rather short, and not all the testers involved in the pilot were independent from the game development process. We also acknowledge that, in this stage, it is not possible to point out which of the factors considered during the development had the most effect on the results.

### Comparison With Prior Work

Mccallum and Boletsis [116] in their literature review of dementia-related serious games reported a proliferation of cognitive training, exercise, and social games targeting dementia as well as its various symptoms. They conclude that serious games for dementia have a real effect on PwD, but the field is still “unchartered.” Robert and colleagues [117] recommend that serious games, adapted specifically for PwD, may constitute an important tool to maintain autonomy. Kenigsberg and colleagues [118] elaborate saying that “by providing pleasurable activities and person empowerment, these games are a way to enter the homes of PwD through technology, to structure collaborative care knowledge related to dementia and to educate stakeholders so they can cope with critical situations in everyday life.” Establishing links between behavioral disorders and their causes could help a personal or virtual coach in developing a care plan and lifestyle training. They close by stating, that the role of technology in improving sensory impairments and facilitating activities of daily living and providing positive experiences is underexplored. Our work is based on these previous studies and recommendations and focuses primarily on facilitating activities of daily living and providing positive experiences for PwD. This area has not been hitherto sufficiently researched.

### Conclusion and Future Work

Based on both qualitative and quantitative analyses, our pilot, proof of concept study demonstrates that our game was acceptable, accessible, enjoyable, and engaging for PwD. We believe that this type of game set may be useful in creating activities for people with moderate to advanced dementia. These types of serious games may provide meaningful activities for the dyad—PwD and the caregivers of PwD. Such games may also be a good way to assess cognitive status of PwD in a nonthreatening way [119-123]. Future work should also consider cultural and language aspects that may affect performance and engagement (for a discussion, see [124]), as well as aspects of the testers themselves [125].

The significant improved speed for task completion may also suggest that the theoretical methodology used in constructing the game screens is suitable for PwD as it utilizes their remaining capacities - implicit memory and stimulates learning. Our future goal is to expand the game activities based on our holistic theory driven matrix. We aim to add more game screens and be able to study the transferability effect from game screens to functionality in real life scenarios. We plan to develop a training manual for professional and family caregivers related to how to use the game and deploy the package in a large practical trial with PwD living in the community setting. Finally, to test the game’s efficacy, we wish to evaluate, through a randomized trial, the trajectories of functionality in people with moderate to advanced dementia and the impact of playing the game on this trajectory.

## Abbreviations

ADL: activities of daily living
HCD: human centered design
ISO: international standardization organization
ICT: information and communication technology
MCI: moderate cognitive impairment
MOCA: Montreal Cognitive Assessment
MMSE: mini mental state examination
PwD: people with dementia
